# Non-Vocal Behaviors Are More Frequent During the Decisive Negotiation Phases in Barn Owl Siblings

**DOI:** 10.3390/ani10101777

**Published:** 2020-10-01

**Authors:** Amélie N. Dreiss, Andrea Romano, Raphaëlle Flint, Sarah Bates, Aurélie Vermunt, Isabelle Henry, Charlène A. Ruppli, Alexandre Roulin

**Affiliations:** 1 Department of Ecology and Evolution, University of Lausanne, 1015 Lausanne, Switzerland; Raphaelle.Flint@iucn.org (R.F.); sarah.bates@unil.ch (S.B.); vermunt.aurelie@sfr.fr (A.V.); isabelle.henry@chauves-souris.ch (I.H.); charlene.ruppli@unil.ch (C.A.R.); alexandre.roulin@unil.ch (A.R.); 2 Department of Environmental Science and Policy, University of Milan, 20133 Milan, Italy

**Keywords:** negotiation, multiple signals, gesture, behavior, turn-taking, *Tyto alba*

## Abstract

**Simple Summary:**

Animal communication can involve the use of multiple types of signals. While vocal communication has been widely studied in natural populations, there is a dearth of knowledge about the possible role of vibrations or noises made by body movements in communication processes. By using experimental settings both under natural conditions and in the laboratory, we showed that barn owl nestlings (*Tyto alba*) produced various non-vocal noises. Movement noises were particularly frequent when nestlings were involved in the intense vocal interactions they use to negotiate the priority for access to the next food item delivered by parents. Body movements might therefore have a role in reinforcing vocal signals during competitive interactions among siblings.

**Abstract:**

Animals produce vibrations or noises by means of body movements, which can play a role in communication. These behaviors enhance signal transmission or receiver attention and could be specifically used during turn-taking phases of a reciprocal exchange of signals. In the barn owl *Tyto alba*, nestlings vocalize one after the other to negotiate which individual will have priority access to the impending prey item to be delivered by the parents. Owlets adjust their vocalization to their own hunger level and to their siblings’ vocalization, withdrawing from the contest in front of highly vocal, and hence hungry, motivated nestmates. As sibling negotiation is a multicomponent display, we examined whether body movements could also be part of the negotiation process. To this end, we analyzed whether the vocalizations of one nestling affected its nestmate’s movements in three separate experiments: in natural nests, in the lab, and using a playback procedure. Nestling barn owls move in a variety of ways, such as repeated tapping of the floor with a foot, scratching the floor with claws, or flapping wings. Body movements were more frequent during the turn-taking phases of vocal interactions, when siblings emitted longer calls and at a greater rate. Once an individual monopolized vocal activity, siblings became less vocal and less active. Moreover, owlets produced more noisy body movements during the phases of vocal interactions which are crucial to prevail in negotiation. Non-vocal physical activities might reinforce vocal signals during sibling to sibling (sib–sib) interactions, or reflect owlets’ arousal, in the critical period during which they vocally settle which individual will dominate the competition.

## 1. Introduction

A signal is a display emitted by an individual, eliciting a response from one or several receivers, and that has evolved for this effect [1]. Signals can be conveyed by several modalities, most notably acoustic, such as vocal sounds, visual, such as color patterns, or olfactory. In fact, communication often simultaneously or sequentially combines several types of signals and modalities, and some signals are often accompanied by particular behaviors [2,3,4,5,6]. These behaviors sometimes enhance the signals, allowing the receiver to more rapidly detect a conspecific or assess a mate more accurately [3,4,5,6]. Although body movements—tapping or drumming with a part of the body on a surface—are not necessarily considered to be signals, they can have a non-negligible role in communication [7]. Such behavior is observed in humans, who often use gestures while speaking. Sometimes our gestures carry communicative functions [7] and enhance speech processing [8]. They can also be a by-product, potentially giving a cue to receivers, but without having evolved to transmit a message. For example, a person tapping on a table produces a sound which can mean that he or she is nervous [9]. In animals, a large number of vertebrates and arthropods produce substrate-borne vibrations that are perceived and can be used to communicate with a mate or group members, or used as a cue of presence by predators or conspecifics [10]. Vibrations, or seismic signals, influence the processing of visual signals in the jumping spider *Habronattus dossenus* [11], or can be used as an alternative to calling when other channels are disturbed with interferences, such as in the white-lipped frog *Leptodactylus albilabris*, whose males produce impulsive thumps in intraspecific communication [12]. In elephants, seismic signals are considered an important complement to their major acoustic and visual communication modes (review in [13]). In addition, several bird species have been observed to use non-vocal acoustic signals produced with the bill, feathers, or feet, in the context of courtship [14,15,16,17,18].

The begging behavior of altricial species is typically a multimodal communication system, with nestling birds, pups, and insects using body postures, color patterns, and vocalizations to compete for parental feeding [19,20]. Although begging behavior has been primarily studied in the context of parent-offspring conflict and sibling competition, offspring can also produce begging behaviors in the absence of parents [21,22,23]. This behavior has been studied extensively in the barn owl *Tyto alba* in which owlets not only beg towards their parents, but also vocally interact all night long with their nestmates [24]. Each nestling can produce thousands of calls per night while parents are foraging far from the nest. Sibling to sibling (sib–sib) vocal interactions in the absence of parents, hereafter referred to as “sibling negotiation”, predict which owlet will be fed at the next parental feeding visit [24]. The hungriest owlet vocalizes more than its less hungry siblings, and hence it signals its motivation to monopolize the prey item next delivered by a parent, which deters its siblings from competing [25,26,27]. In practice, the end of a negotiation session is settled by the arrival of a parent with a food item. At this point, the sibling that has dominated the negotiation process will beg the parent for food, while the others refrain from fiercely competing. A number of conditions that pertain to the evolution of sibling negotiation apply to the barn owl system [28]. Sibling physical competition being energetically expensive, time consuming and potentially dangerous by attracting predators, barn owl siblings would benefit substantially from the non-aggressive resolution of a contest through vocal negotiation rather than by fighting. Parents bring one food item per visit, which is indivisible and hence consumed by a single offspring. For this reason, only one individual is paid back for the investment in sibling competition. In these circumstances, and because the food need substantially varies among siblings, nestlings have strong incentives to assess how hungry they are in relation to their siblings. When their siblings are particularly hungry, nestlings benefit from momentarily withdrawing from a contest, because the likelihood of monopolizing a prey item is low. Finally, sibling negotiation, as cooperation, is more likely to evolve in systems in which individuals are highly related [28,29]. The rate of extra-pair fertilization is very low in the barn owl, with only 2% of the nestlings being sired by a different male than the one that feeds them [30].

As in any negotiation process, participants invest in negotiation signals according to how they value the coveted resources (in the barn owl, the prey brought by parents), which is directly related to their food needs and body condition. The situation is however more complex. Although between two feeding events and in the short-term (e.g., about several minutes) the need for resources of each participant does not vary, the negotiation process is still very dynamic, being sensitive to the social interaction process itself. Accordingly, barn owl siblings adjust their vocal negotiation to one another on a short-term basis, independently of variation in food need, as shown by observational and experimental studies [27,31,32,33]. The negotiation process between siblings may be similar to human dialogues, with specific turn-taking rules being used to decide when an individual will start or stop calling [31,32,34]. When an individual starts calling, it gradually produces longer but fewer calls per minute. When it “relaxes” and makes shorter calls, with longer pauses, another sibling is likely to interrupt it by emitting short calls and can gradually monopolize the negotiation [31,32,34]. By following these turn-taking rules, individuals have the opportunity to alternatively signal their motivation and perceive the motivation of their counterparts, which allows each individual to adequately decide to withdraw from the contest or to pursue competitive interaction [31,32,34].

In humans, body movement is important in turn-taking [35,36], but in non-human animals its role remains understudied. The lizard *Anolis sagrei* may use head nods in a conversational turn-taking style during communication [37]. In the present study, we investigated whether the different body movements observed in barn owl nestlings (tapping with beak and feet, scratching with claws, and other body movements that make noises) are part of the sibling negotiation process. We investigated whether body movements, defined as non-vocal physical activity, occur at certain phases of sibling-to-sibling vocal interactions and whether they are associated with hunger level, sex, age, and position in the within-brood age hierarchy. In a first experiment, we investigated the types of body movements performed in experimental two-chick broods video-recorded in their natural nests 15 min before the first parental visit of the night. We tested whether body movements were associated with call rate, an important component of sibling negotiation. In a second experiment in the laboratory, we analyzed interactions between two siblings that were physically separated but allowed to interact acoustically. Over two successive nights, the nestlings were alternatively food-deprived and food-satiated. In this study, we analyzed noise produced by body movements in relation to call rate and duration of calls, a second important component of sibling negotiation. This second experiment in controlled conditions and with a larger sample size allowed us to confirm the results obtained in natural nests. In a third experiment, we tested how single owlets respond to playback with respect to their own vocalizations and body movements. The playback was a pre-recorded dialogue between two owlets in which the calls of one of the owlets were deleted. This experiment is important to test whether the calling behavior of an individual influences the noise made by a sibling.

If body movements are associated with individual level of need, along with call duration and call rate [27], we would expect them to be more frequent in food-deprived than food-satiated nestlings (experiments 1 and 2). More frequent movements in food-deprived nestlings can also reflect the physical competition, as hungry nestlings tend to try to get closer to the nest entrance to gain precedence in food delivery [25]. Under this hypothesis, we would also predict that within a brood the youngest nestlings would move more, because they usually put more effort into sibling competition than older nestlings [38]. By examining the direction of the relationship between body movements and vocal behaviors, we finally wished to explain whether body movements may interfere with (negative correlation) or, alternatively, possibly reinforce (positively correlation) vocal sibling negotiation.

## 2. Materials and Methods

### 2.1. Study Organism

The study was performed in western Switzerland (46°4’ N, 6°5’ E), in a natural population breeding in nest-boxes (62 × 56 × 37 cm) mounted on barns. Barn owls are monogamous within each reproductive season with very little extra-pair paternity [30]. Females lay up to 11 eggs, approximately one every 2.5 days sequentially and, because incubation starts after the first egg has been laid, hatching is staggered. As soon as nestlings are sufficiently thermo-independent and able to swallow a whole prey item or rip it into pieces without maternal help (at ca. 3 weeks of age), the parents leave them on their own in the nest most of the time, except for the periodic feedings, which occur throughout the night. At each feeding visit, a parent brings a single food item per nest visit, occurring on average every 45 min, usually a small rodent, to only one of its offspring. The owlet is fed this way for the first 50 to 70 days of its life, at which time it leaves the nest. The age of the nestlings was estimated by measuring the flattened wing from the bird’s wrist to the tip of its longest primary, which has been shown to be a reliable proxy for age [39]. Nestling sex was determined using molecular markers [40]. Considering that there is wide knowledge that the sex is not related to negotiation calls, nor to its behavioral effects on siblings [27,32,34,41,42,43], pairs of senior-junior nestlings (see below) were randomly composed with respect to sex.

We recorded vocal and non-vocal behaviors in three experiments in 1997, 2008, and 2009.

### 2.2. Experiment 1—Video Recording of Nestling Pairs in the Field

In 1997, we recorded behaviours in 13 pairs of nestlings (14 males and 11 females, 1 unsexed) during the 15 min preceding the first parental feeding visit of the night, using an infrared video camera. On the video footage, we could classify the various movements performed, and count the emitted calls. However, considering that calls are usually very short, we could not accurately measure the duration of each call emitted using videos. We created two-chick broods by temporarily removing all but two randomly chosen nestlings from the nests (initially the studied broods contained two to nine nestlings, mean ± s.e.m.: 4.7 ± 0.2). The oldest nestling, referred to as “senior”, was on average 36 days old (range = 20–54) and the “junior” sibling on average 31 days (range = 17–52). These 13 pairs were recorded on three successive nights. During the daylight hours before recording, from 09:00 to 21:30, we kept each of the two siblings in a ventilated plastic box of the same size as the nest boxes with either three dead mice (food-satiated treatment) or without food (food-deprived treatment). We note that the food-deprived group represents the natural condition for nestlings occurring every day before parents start to deliver food to the nest. One night the senior was food-satiated; on another night the junior was food-satiated, and on a third night the two siblings were food-deprived. The order of the manipulations was randomized across the three nights (for further details see [43]). At 21:30, we put the two individuals back in their nest-box to record their behaviour until approximately midnight. The present experiment is based on the same video recordings used in previous studies for which the aim was to investigate the role of vocalizing on obtaining prey [25,43].

### 2.3. Experiment 2—Acoustic Recording of Nestling Pairs in the Laboratory

In 2008, we analyzed the dynamics of nestling body movements for a longer period of time (ca. 270 min, from 19:00 to 23:30) in 52 pairs of wild nestlings from 34 broods recorded in laboratory conditions. In contrast to the first experiment, we not only counted calls but also measured their duration. Because we did not video-record nestlings, body movements were detected on audio recordings. Indeed, they can be inferred from visible waveforms on Audacity. Then, the observer listened to the sound to assess what kind of sound it was (see below for details and Supplementary Materials for examples).

Nestlings, aged between 24 and 46 days (mean ± SD = 34.8 ± 5.0), were placed in nest boxes similar to those in which they hatched (100 × 60 × 50 cm; see [27]) but with a thin plywood wall pierced by holes, allowing us to physically separate the two siblings. In this way, the oldest nestling of a pair (on average 37 days old, range = 25–45) and its “junior” sibling (32 days on average, range = 24–43) could interact audibly but not visually or physically. This procedure was used to investigate the potential role of acoustic communication on sibling negotiation independently of physical competition. Vocal and non-vocal noises were recorded using Beyerdynamic microphones (MC930, Beyerdynamic GmbH & Co KG, Heilbronn, Germany). In order to distinguish between the two nestlings under investigation, two soundtracks from two microphones oriented in opposite directions, each facing one individual, were recorded. We then compared the signal level and timing from the two microphones to assign sounds to one or the other owlet. The young were brought during the afternoon and held for three nights, the first of which was used for acclimation (thus, no data were collected during this night). The second and third nights, nestlings were randomly assigned to one of two feeding treatments. In the morning, “food-satiated” nestlings were provided with mice (*Mus musculus*) ad libitum until 16:00 when any remaining mice were removed; each individual consumed on average 87 ± 2 g of mice. Nestlings of the “food-deprived” treatment were visited as often but were not offered food. If one individual had been food-satiated on the second night, on the third night it was food-deprived, and vice versa.

### 2.4. Experiment 3—Playback Experiment

In 2009, we carried out a playback experiment to test the causal effect of vocal negotiation on nestling physical behavior. We brought 56 nestlings to the laboratory (36 females and 20 males; aged 27 to 46 days old) from 16 different wild broods. The owlets were placed in a soundproof wooden nest box similar to the one used the previous year, but the individuals were placed alone in the box containing a loudspeaker (near05experience, ESI Audiotechnik GmbH, Leonberg, Germany) placed on the other side of the wooden divider (for further details see [27]). To build playback soundtracks, we selected five recordings from those gathered in 2008 with the criterion that one of the two recorded chicks never stopped calling for more than 3 min. The calls of the less voluble nestling and all noises were then deleted from the soundtrack using the software Audacity 1.3 beta freeware, so that we broadcasted calls only of the most vocally active individual. The five selected individuals were two females and three males, aged 27 to 41 days old (mean ± s.e.m: 35 ± 2). A microphone (MC930 Beyerdynamic GmbH & Co KG, Heilbronn, Germany) was also fixed on the ceiling of the box to record the vocal and body movement response to the playback of the singleton nestling. The recordings were broadcasted from 00.30 a.m. and lasted between 73 and 94 min.

### 2.5. Body Movements

On video footage for experiment 1, A.L. assessed different types of body movements: displacement (walking), feet movement (tapping with the foot on the floor and scratching the bottom of the box with claws), flapping the wings, self-preening and scratching. On audio recordings, non-vocal audible noises were screened using Audacity 1.3 by S.B. for experiment 2 and R.F. for experiment 3. A few examples of noises and their interpretation in terms of likely associated behavior are given in Supplementary Materials files 1, 2 and 3. When different types of body movements were assessed using audio recordings, in a small proportion of cases (ca. 2%) it was not possible to correctly establish which movement type was producing the noise. In addition, in some other circumstances the noise was produced by two or more concomitant (or temporally very close) movements (e.g., scratching and wing flapping). In this latter case, we classified the noise as the movement which had the longest duration. However, and importantly, sounds made by body movements are clearly distinguishable from vocal calls or ambient noises.

The presence/absence of body movements recorded on video footage or audio recordings was assessed each minute. Each year was analyzed by a different observer, which may explain, at least in part, the difference in detection rate between experiments. Indeed, nestlings were recorded performing a body movement in 66% and 52% of recorded minutes in the field (experiment 1) and when facing a playback (experiment 3), respectively, while only in 7% of minutes for nestling pairs in the laboratory (experiment 2). Vocal behaviors, i.e., call rate and call duration (the latter only in experiments 2 and 3), were measured in previous studies [27,31,44].

Using videos of experiment 1, we estimated the frequency of each body movement and if different movements were independent from each other by measuring whether any body movement occurred in each minute (yes or no). Owlets spent a lot of time walking inside the nest-box (53% of minutes), and performed other noisy body movements less often, like scratching the ground (1% of minutes), tapping a foot on the ground (8%), flapping wings (10%) or self-preening and scratching (33%). The different types of body movements were positively correlated with each other. For instance, nestlings were more likely to tap the ground with their foot if during the same 1 min interval they were scratching the floor (effect of tapping likelihood on the likelihood of scratching in a generalized mixed model with as random factors individual and video recording nested in brood: F_1,902_ = 4.07, *p* = 0.04). When nestlings walked, they also scratched their foot and flapped their wings during the same minute 47% and 16% of time, respectively; they showed these behaviors only 21% and 2% of the time when they stayed still (effect of walking on scratching: F_1,901_ = 32.8, *p* = 0.0001; effect of walking on wing flapping: F_1,886_ = 36.09, *p* = 0.0001). Because the probability of performing a given body movement was positively associated with the probability of doing another type of body movement, for each minute we determined whether nestlings performed at least one category of body movement and we used this variable in the statistical analyses.

### 2.6. Statistical Analyses

Each minute, we measured call rate and assessed whether any body movement occurred (yes or no). This latter binary variable was used because (1) body movements were correlated with each other and (2) on acoustic recordings, it was not possible to verify that the classification of the different body movements was always correct (see above). However, separate analyses (details not shown) for each type of body movement (tapping, scratching the floor, flapping wings and walking) gave very similar results. We fitted generalized mixed models with binomial distribution and logit link. For each pair of nestlings, one individual was randomly selected to be analyzed, in order not to consider the same interaction twice.

For experiment 1, the likelihood of performing body movements was tested according to the body movements and the call rate of the vocalizations of the partner sibling, as well as the call rate of the vocalizations of the nestling under scrutiny. In addition, sex, age, a dichotomic factor indicating whether the nestling under scrutiny was junior or senior, the food treatment of both nestlings, and the time elapsed from the beginning of the videotape were included as additional predictors. Moreover, considering that variation in nestling behavior according to own and siblings’ vocal calls might not always be linear (e.g., [41]), we also tested the quadratic effect of the call rate of the vocalizations of both siblings. Finally, individual nestling identity as well as the video recording nested in the brood were set as random factors.

For experiment 2, the likelihood of performing body movements was tested according to the same predictors used for experiment 1 with a single difference. Since we focused only on a single nestling per pair, we only included the food treatment of the nestling under scrutiny as an additional predictor. In this model, brood and individual identities nested in the pair of siblings were included as random factors.

For the playback experiment (experiment 3), the likelihood of performing body movements was tested according to the call rate of the vocalizations emitted by the playback and those emitted by the nestling under scrutiny. We again included nestling’s sex and age, as well as the time elapsed from the beginning of the videotape as additional predictors. Brood and individual identities nested in playback sequence identity were set as random factors.

Two additional analyses were performed for experiments 2 and 3 [27] in order to assess the relationship between the likelihood of performing body movement and nestlings’ call duration, which was not measured for experiment 1. We first ran a generalized mixed model with binomial distribution (i.e., at least one detected movement or no detected movements), with the same independent terms as in the above models, plus the call duration of the nestling under scrutiny (average of all calls emitted within each minute), and its quadratic term to test for non-linear relationships. In a second model, we added partner’s (live nestling or playback) call duration and squared call duration as additional predictors. The own call duration and the partner call duration were not added in the same model, because it would restrain the analyses to the minutes when both individuals called (when no call was emitted in a given minute, there are no data for mean call duration). The results are, though, similar if own call duration and partner call duration are in the same model. The random factors were the same as in models above.

For each model, we did not perform selection of terms, but removed non-significant interaction terms. All the analyses were performed by using SAS v.9.1 (SAS Institute Inc., Cary, NC, USA).

### 2.7. Ethical Note

On video footage we did not notice any sign of distress to either the nestlings or adults. The experiments had no detrimental effect on the nestlings, as shown by the absence of a rise in baseline corticosterone level compared to undisturbed conditions [31,45]. Birds were returned each time to their original nest in the wild at the end of the experiments. The original nest was always left occupied by at least one owlet to prevent parents abandoning their nest. Mice for feeding in the laboratory were euthanized by CO_2_ and bought frozen from an animal house (Reptiles Farm, Servion, Switzerland). The experiments were approved by the veterinary services of the Vaud canton (form no. 2109.1).

## 3. Results

**Figure 1:**
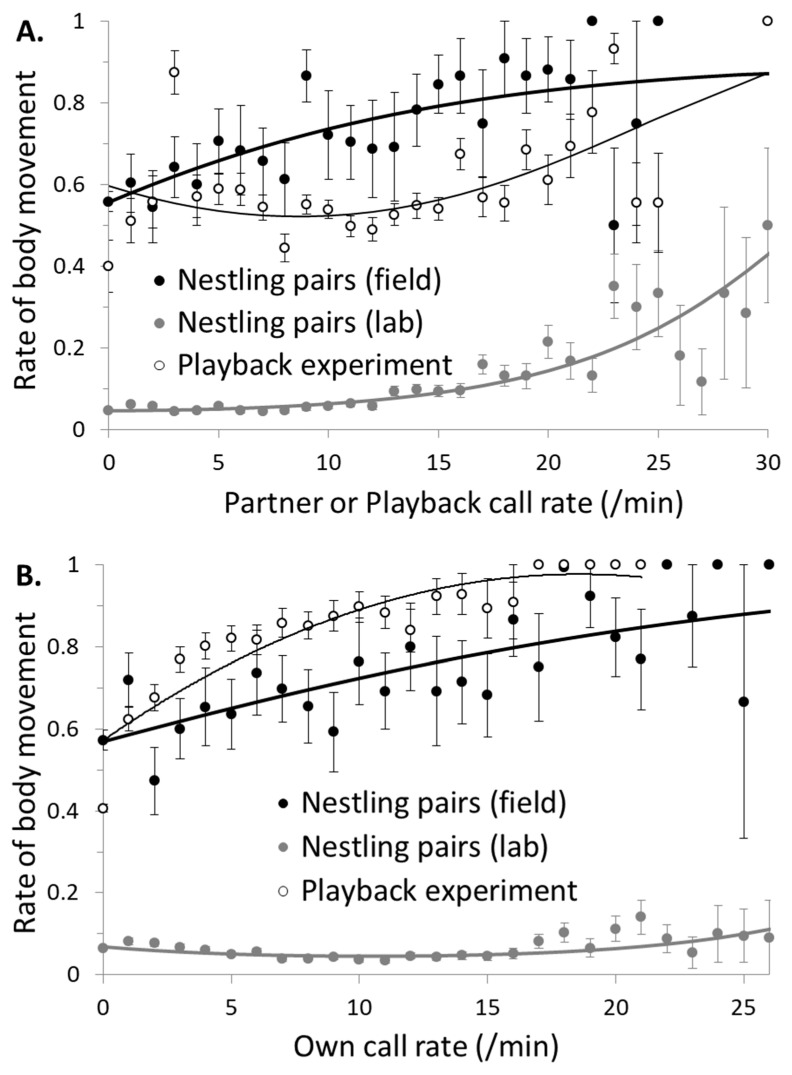
Body movements (occurrence in each minute) of focal barn owl nestlings according to (**A**) partner’s call rate and (**B**) nestling’s own call rate. Behaviors were recorded in pairs of nestlings in their natural nest (black symbols, experiment 1) or in the laboratory (grey symbols, experiment 2), but also in singleton nestlings to which playback calls were broadcast at different rates (open symbols, experiment 3). Bars represent standard errors and lines are estimates of the models. The different types of body movements were not differentiated.

**Figure 2:**
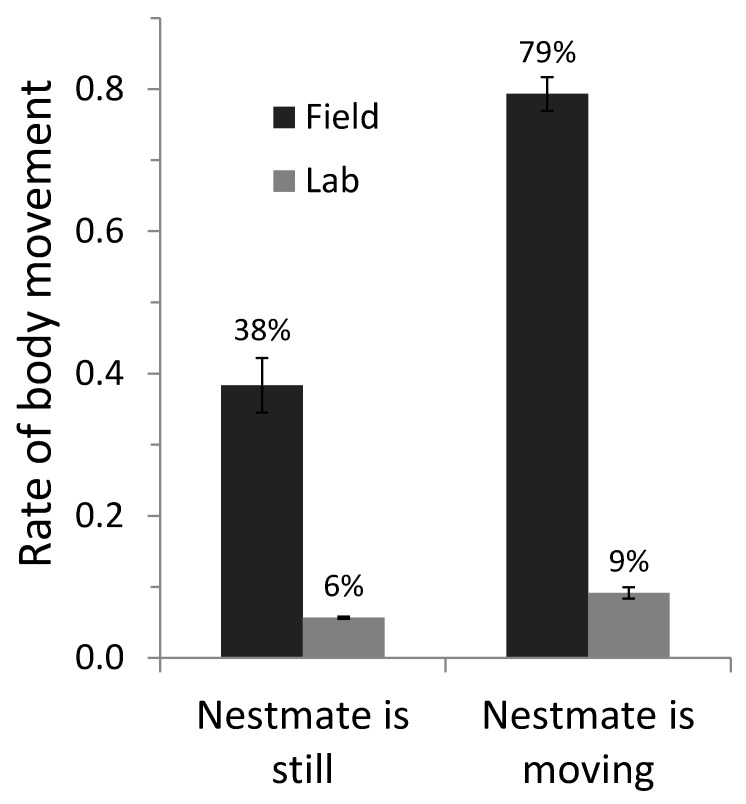
Body movements (occurrence in each minute) of focal barn owl nestlings according to whether their partner was still or performing a body movement, in pairs of nestlings in their natural nest (black bars, experiment 1) or in the laboratory (grey bars, experiment 2). Bars represent standard errors.

Owlets were more likely to move their body when their partner was more vocal (Figure 1A, Table 1, effect—partner/PB call rate) and physically more active (Figure 2, Table 1, effect—partner body movements). The relationship between the probability of a focal nestling moving its body and the nestmate’s call rate (experiments 1 and 2) was confirmed in the playback experiment (experiment 3). Indeed, singleton nestlings were more likely to engage in body movements when the loudspeaker emitted more playback calls (Table 1, Figure 1A).

Owlets also performed more body movements when they called more, in the field or when responding to a playback (Figure 1B, Table 1a,c, effect—call rate). This effect was however not detected in pairs of nestlings in the lab, for which the non-vocal behavior was minimal at medium call rate (i.e., at ca. 10 call/min, see Figure 1B), as shown by the effect—call rate ^2^ (Table 1b).

**Figure 3:**
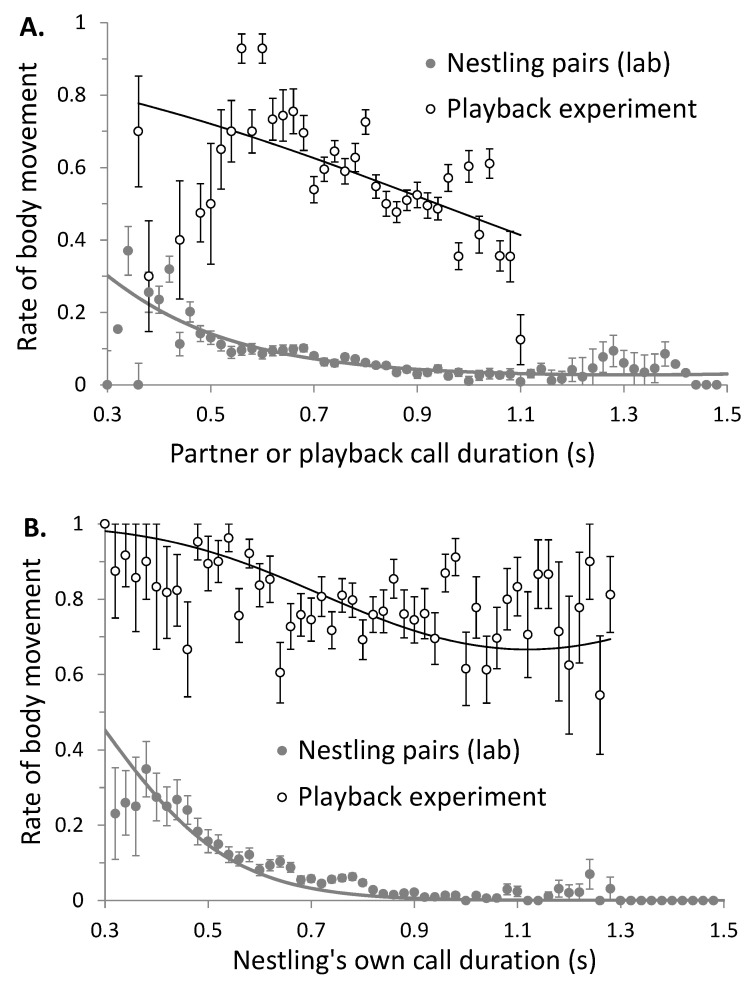
Body movements (occurrence in each minute) according to the duration of negotiation calls performed by the (**A**) partner and (**B**) the focal barn owl nestling itself. Behaviors were recorded in pairs of nestlings in the laboratory (grey symbols, experiment 2) and in singleton nestlings to which we broadcast playback calls of different durations (open symbols, experiment 3). Bars represent standard errors and lines are estimates of the models.

In contrast, nestlings moved less often when their own calls and the calls of their partner were longer (Figure 3). This result was found consistently in pairs of nestlings in the laboratory (Table 1b) and in owlets responding to a playback (Table 1c). Body movements did not vary according to food treatment of focal nestlings and partners (Table 1a,b). It was not related to absolute age in days or seniority (junior vs. senior), but marginally decreased over the night. Males performed slightly more body movements than females in experiment 3 (Table 1c), but not in others (Table 1a,b).

## 4. Discussion

We found that in barn owl nestlings, body movements were performed non-randomly in relation to the emission of vocalizations. Nestlings produced more noisy movements when they, and their siblings, produced more calls and when their own calls and those of their siblings were shorter. These results were confirmed in a playback experiment: the owlets adjusted their body movements in the same way in relation to the variation in the number and duration of the playback calls. In the barn owl, the negotiation process is temporarily settled once an owlet is the only one to produce calls. At this point, this vocally dominant owlet gradually spaces the timing of call emission and increases its call duration [31,32,34]. Conversely, before one owlet vocally dominates, when two owlets are calling during the same minute, their call duration and call rate are, respectively, positively and negatively adjusted [31,32,34]. This new study shows that body movements are particularly prevalent in this phase of intense exchange of numerous short calls. On the whole, our results show that owlets produced many non-vocal noises during periods of high vocal competition, while once a nestling has succeeded in becoming vocally dominant, it produced longer calls but more spaced in time and it made fewer noisy body movements.

The dynamics of vocal negotiation and body movements were generally positively correlated. Therefore, movement noises, made by tapping, scratching with a foot, walking or flapping wings, and vocal calls are not competing activities that nestlings could not perform simultaneously. A possible explanation for this positive correlation between vocal calls and non-vocal noise is that body movements might reinforce the negotiation processes. Under this interpretation, the body movements would have an enhancing effect on call rate [5,6], as a way to get attention and to enhance the transfer of information. We can, for example, compare this with humans using gaze instead of words in a conversation to coordinate turn-taking [36]. In addition, this finding clearly indicates that non-vocal noise does not interfere with the transmission of vocal signals. The sound produced by body movements are on average shorter and softer than the negotiation calls (A.D., pers. obs.), hence they do not blur the emitted calls, but could act as an additional signal.

With the present data we cannot properly investigate whether movement noises would constitute an alternative way of communicating to vocal calls, because we did not test the direct effects of non-vocal noise on siblings’ behavior alone. This issue therefore represents a drawback of our study. However, our results suggest that body movements and calls are probably not interchangeable. On the one hand, vocal signals are well known to be crucial to convey information about individual motivation to compete for food, and to determine specific behavioral responses in siblings (e.g., [25,27,31,32,33]). On the other hand, the present study shows that body movements are not related to the experimentally manipulated food availability, thus indicating that noise, differently from vocal calls, does not signal individual hunger level. Body movements were not related to the age hierarchy, while juniors are known to compete more intensely for food [38]. It is therefore unlikely that non-vocal noise alone can convey reliable information about the level of motivation to compete and induce a variation in siblings’ behavior. However, adding a component to the negotiation on top of the production of calls may improve detectability, discriminability, and memorability by nestmates [46]. In Partan and Marler’s classification of multimodal signals [5,6], vocal calls and body movements (in case the latter ones would alone elicit a response in the receiver) could be therefore considered non-redundant signals because they do not convey the same information independently. Future specific experimental studies will be helpful to understand whether the nestmates’ response to both vocal calls and movements is increased (i.e., a case of modulation in the Partan and Marler’s classification) or unchanged (i.e., a case of dominance in the Partan and Marler’s classification) compared to vocal calls alone.

Alternatively, body movements may simply be a way to heighten nestling’s presence or signal its arousal. Barn owls have broods of multiple nestlings, which could have provided the ideal environment for a multicomponent negotiation process to evolve, given that complex interactions between siblings can be expected to play a significant role in shaping complex behaviours. It would be interesting to compare the negotiation behaviour between nestlings of different brood sizes, to see if different signal components are used in different social environments. Finally, we also cannot exclude the possibility that body movements are not part of the sibling-to-sibling communication process, but that they are related to the particular nestling condition during critical phases of the vocal interactions.

All body movements being correlated, we could not disentangle whether each movement has a different function. Tapping, drumming, or “step-dancing” with feet or wings has been observed in several bird species as a component of courtship display for instance, but only in the context of sexual selection [15,17] and not in the context of sibling competition. With additional playback experiments or mechanical/video models, it would be possible to accurately test the behavioural response of nestmates to the different types of body movements. The next step would therefore be to experimentally test the response of siblings to different body movements. This would enable us to establish a clearer distinction between noises forming part of sibling negotiation and those specifically used for other functions.

Finally, we admit that the difference in movement rate between experiment 2 and the others might indicate that the detection rate of movements using audio was lower than using video. However, we note that, with the single exception of the lack of the significant effect of own call rate on own body movements in experiment 2, which is conversely significant in experiments 1 and 3 (see Figure 1B), all the results are consistent among the experiments. This observation thus indicates that, while the absolute movement rate might have been affected by the experimental setup, the relative difference in movement occurrence among the different phases of negotiation is maintained. Such consistency in the results obtained using different procedures thus indicates that our main results are rather robust and not affected by the sampling procedure.

## 5. Conclusions

This study underlines the complexity of animal communication systems. Although most work focuses on one modality, many species use multiple signals when communicating [47,48]. We show here that even behaviors that seemed unrelated to communication at first sight, such as moving, flapping wings, or scratching, were in fact highly correlated with vocal communication and might be used during information exchange processes.

## Figures and Tables

**Table 1:** Nestlings’ likelihood of performing body movements, according to their own vocal behavior, food treatment, and characteristics and the vocal behavior and food treatment of their nestmate (live partner or playback). Three settings are analyzed: (a) nestling pairs in their natural nest, (b) nestling pairs in the laboratory, and (c) singleton nestlings responding to a playback. Result of generalized linear models. Superscript numbers indicate quadratic terms (see main text).

	a. Nestling Pairs in the Field (Experiment 1) d.f. = 417			b. Nestling Pairs in the Lab (Experiment 2) d.f. = 12,251			c. Playback (Experiment 3) d.f. = 4372		
Effects	F	*p*	Estimate	F	*p*	Estimate	F	*p*	Estimate
Partner body movements	12.9	0.0004	1.106 ± 0.308	11.1	0.0009	0.523 ± 0.157	-	-	-
Call rate	15.2	0.0001	0.126 ± 0.032	3.5	0.061	−0.038 ± 0.02	272.1	<0.0001	0.561 ± 0.034
Call rate ^2^	0.9	0.34	0.004 ± 0.004	6.7	0.009	0.003 ± 0.001	83.2	<0.0001	−0.024 ± 0.003
Partner/Playback call rate	15.0	0.0001	0.276 ± 0.071	124.9	<0.0001	0.118 ± 0.011	4.4	0.037	0.058 ± 0.028
Partner/Playback call rate ^2^	6.2	0.013	−0.008 ± 0.003	1.7	0.19	0.001 ± 0.001	5.4	0.019	0.003 ± 0.001
Sex (male vs. female)	1.0	0.31	−0.773 ± 0.767	0.2	0.67	0.114 ± 0.267	5.2	0.022	0.483 ± 0.211
Age	0.4	0.56	0.039 ± 0.067	0.7	0.41	−0.023 ± 0.029	0.7	0.40	0.024 ± 0.028
Seniority (junior vs. senior)	1.1	0.30	0.786 ± 0.758	1.0	0.31	0.303 ± 0.3	-	-	-
Food treatment (food-deprived vs. satiated)	0.0	0.99	−0.005 ± 0.775	0.0	0.93	−0.009 ± 0.103	-	-	-
Partner food treatment (food-deprived vs. satiated)	0.0	0.92	0.078 ± 0.735	-	-	-	-	-	-
Time (min)	0.2	0.67	−0.013 ± 0.031	10.5	0.001	−0.004 ± 0.001	157.6	<0.0001	−0.018 ± 0.001
				d.f. = 7024			d.f. = 1653		
Call duration	-	-	-	11.2	0.0008	−6.5 ± 2.3	22.7	<0.0001	−11.0 ± 2.3
Call duration ^2^	-	-	-	6.0	0.014	−5.3 ± 1.6	13.5	0.0003	4.9 ± 1.3
				d.f. = 7068			d.f. = 4312		
Partner/Playback call duration	-	-	-	50.0	<0.0001	−12.3 ± 1.7	0.7	0.41	2.6 ± 3.1
Partner/Playback call duration ^2^	-	-	-	12.0	0.0005	3.9 ± 1.1	4.6	0.031	−4.1 ± 1.9

## Data Availability

The datasets analyzed during the current study are available from the corresponding author on request.

## Supplementary Materials

The following are available online at https://www.mdpi.com/2076-2615/10/10/1777/s1, A few examples of noises and their interpretation in terms of likely associated behavior are given in Supplementary Material files 1 (flapping), 2 (repeated-tapping), and 3 (scratching and calls).
